# Interface slip of steel–concrete composite beams reinforced with CFRP sheet under creep effect

**DOI:** 10.1038/s41598-022-27023-y

**Published:** 2022-12-26

**Authors:** Xiangyang Jian, Ni Zhang, Haiqing Liu, Zhongwei Zhao, Ming Lei, Zimu Chen

**Affiliations:** 1 School of Civil Engineering, Liaoning Technical University, Fuxin, 123000 China; 2 China Construction Fifth Engineering Division Corp., Ltd, Changsha, 410004 China

**Keywords:** Civil engineering, Mechanical engineering, Structural materials

## Abstract

Under the creep action of composite steel and concrete beams reinforced by carbon-fiber-reinforced polymer (CFRP) sheet, the face of the CFRP sheet, steel beam, and concrete slab beam produce relative slip. This slip affects the interface interaction, reduces the bearing capacity and stiffness of the members, and increases the deformation. In this paper, elastic and energy methods are used to analyze the interface forces between steel beams and concrete slabs reinforced by CFRP sheeting under the action of concrete creep. The calculation formulas for interface slip, axial force, and incremental deformation are established. The influence of design parameters on the mechanical properties of the interface is analyzed. Results show that the increments in interface slip, axial force, and deformation are zero on the 28th day. With increasing age, the increments in interface slip, axial force, and deformation gradually increase, and the increase is large in the first 100 days; it basically remains unchanged during the time interval from 100 to 1028 days. When the load increases by 5 N/mm (5 kN), the slip increments increase by approximately 0.004 mm, 0.002 mm, and 0.002 mm. The increments in axial force are approximately 19.4 kN, 15.9 kN, and 16.1 kN. The deformation increments increase by approximately 1.7 mm, 1.1 mm, and 0.6 mm.

## Introduction

Steel structures are widely used in industrial and civil buildings and bridge engineering owing to their convenient construction and strong practicability^1,2^. Owing to the influence of various factors, such as use and environment, various defects and damages exist in the steel structure^3,4^, especially when the steel structure is overloaded. In other words, the service load of the structure is much greater than the allowable service load of the structure^5^; this situation accelerates the aging of the structure and reduces its service life, especially when the structure itself has minor damage caused by construction. Overloading increases the damage to the structure, and the microscopic defects gradually expand and converge, resulting in the deterioration of the material in terms of macroscopic mechanical properties; it even causes engineering accidents. Therefore, the study of ways to strengthen and repair the steel structure has always been an important endeavor in civil engineering. Data show that reconstruction projects can save approximately 40% of the investment and shorten the construction period by approximately 50% when compared with new construction^6,7^. Seeking cost-effective steel structure reinforcement and repair technology is not only a technical problem to be solved but also a social problem related to sustainable development.

The traditional methods of strengthening steel structures include increasing the number of sections of steel members, adding additional rods and supports, and prestressing reinforcement. Among them, increasing the number of sections of the steel member involves connecting the new and original steel members by welding, riveting, bolting, or pasting steel plates^8,9^. The structure changes from a plane to a space^10,11^, and prestressing reinforcement is set to prestressed tie rods at appropriate parts of the steel structure to form a stress opposite to the load in the structure^12–14^. To a certain extent, these methods increase the cross-sectional size of the components, increasing the weight of the components and changes in the stiffness. This results in a redistribution of the internal forces of the structure, inconvenient transportation and installation, complicated construction, and high maintenance costs. In recent years, the use of Fiber Reinforced Polymer (FRP) sheets to reinforce steel beams has emerged as a new reinforcement method at home and abroad. This reinforcement method involves pasting or anchoring FRP sheets on steel beams. The high strength of FRP material is used to improve the bearing capacity and stiffness of the beam to achieve the effect of reinforcement.

At present, both domestic and international experts and scholars have studied the interface interaction between steel beams and CFRP sheets^15–36^ and between steel beams and concrete slabs, but studies on interface slip analysis under the influence of the creep effect are scarce. Therefore, based on previous research, the elastic and energy variation methods are used to establish the interface slip increment, axis, and axis of the CFRP sheet-reinforced steel–concrete composite beams under the action of concrete creep. The calculation formulas for the force and deformation increments of the composite beam are discussed, along with the influence of the design parameters.

## Incremental analysis of interface slip

### Calculation of interface slip increment

#### Elasticity method^37^

**Figure 1:**
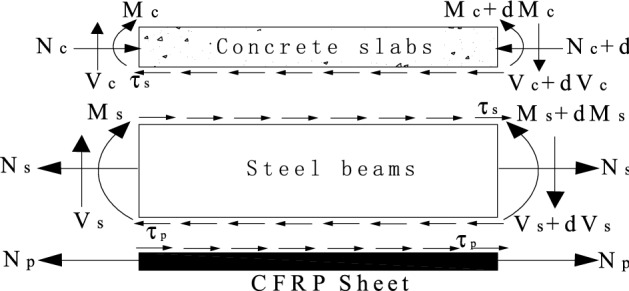
Force graph of unit body.

Since the temperature effect of the steel beam and the creep effect of the CFRP cloth have been given in^38^, only the creep effect of the concrete is considered herein, that is, only the interface force between the concrete slab and the steel beam is considered. Several assumptions are made about the characteristics of steel–concrete composite beam structures reinforced by CFRP sheets^39,42^. Under normal use, the composite beam is an ideal elastic body. The shear connection between the steel beam and the concrete forms a uniform and continuous arrangement along the beam length. Concrete slabs, steel beams, and CFRP sheeting have the same bending curvature before and after deformation, regardless of the vertical lift between them. The cross-section conforms to the assumption of a flat cross-section. The force of the unit body is shown in Fig. 1.

The strain increments on the upper surface of the steel beam and the lower surface of the concrete slab at time $t$ are described below:

1 $$ \Delta \varepsilon_{s} \left( t \right) = \frac{{\Delta N_{s} \left( t \right)}}{{E_{s} A_{s} }} - y_{s} \left( t \right)\Delta \varphi \left( t \right) $$

2 $$ \begin{aligned} \Delta \varepsilon_{c} \left( t \right) & = y_{c} \left( t \right)\Delta \varphi \left( t \right) - \phi \left( {t,t_{0} } \right)\varepsilon_{c} + \frac{{\Delta N_{sc} \left( t \right)}}{{E_{c} \left( {t,t_{0} } \right)A_{c} \left( t \right)}} - \frac{{\Delta N_{c} \left( t \right)}}{{E_{c} \left( {t,t_{0} } \right)A_{c} \left( t \right)}} \\ & = y_{c} \left( t \right)\Delta \varphi \left( t \right) - \phi \left( {t,t_{0} } \right)\frac{N}{{E_{c} A_{c} }} + \frac{{\alpha_{1} M - \beta_{1} N}}{{E_{c} \left( {t,t_{0} } \right)A_{c} \left( t \right)}} - \frac{{\Delta N_{c} \left( t \right)}}{{E_{c} \left( {t,t_{0} } \right)A_{c} \left( t \right)}} \\ \end{aligned} $$

where $y_{s} \left( t \right)$—*t* the distance from the neutral axis of the beam to the top of the beam at time *t*; $y_{c} \left( t \right)$—*t* the distance from the neutral axis of the plate to the bottom of the plate at time *t*; $E_{c} \left( {t,t_{0} } \right)$—effective elastic modulus of the plate adjusted according to the age at time $t$, which can be expressed as $E_{c} \left( {t,t_{0} } \right) = \frac{{E_{c} }}{{1 + \chi \left( {t,t_{0} } \right)\phi \left( {t,t_{0} } \right)}}$; $\chi \left( {t,t_{0} } \right)$—the concrete aging coefficient of the slab calculated from the loading time, *t*_0_, to time *t*, which usually varies between 0.6 and 0.9, and is taken as 0.82^43^; $\Delta N_{sc} \left( t \right)$—sum of the virtual forces of each layer of reinforcement corresponding to the initial strain of the concrete slab, $\Delta N_{sc} \left( t \right) = \sum\nolimits_{i = 1}^{n} {E_{si} A_{si} \phi \left( {t,t_{0} } \right)\left( {\varepsilon_{o} + y_{si} \varphi } \right)} = \sum\nolimits_{i = 1}^{n} {E_{si} A_{si} \phi \left( {t,t_{0} } \right)\left\{ { - \frac{N}{{E_{c} A_{c} }} + \frac{{y_{si} \left[ {M - N\overline{y}} \right]}}{{E_{s} I_{s} + E_{c} I_{c} }}} \right\}} = \alpha_{1} M - \beta_{1} N$; $\alpha_{1} = \sum\nolimits_{i = 1}^{n} {E_{si} A_{si} \phi \left( {t,t_{0} } \right)\frac{{y_{si} }}{{E_{s} I_{s} + E_{c} I_{c} }}}$; $\beta_{1} = \sum\nolimits_{i = 1}^{n} {E_{si} A_{si} \phi \left( {t,t_{0} } \right)\left[ {\frac{1}{{E_{c} A_{c} }} + \frac{{\overline{y}y_{si} }}{{E_{s} I_{s} + E_{c} I_{c} }}} \right]}$; $\phi \left( {t,t_{0} } \right)$—concrete creep coefficient of the slab^40^, $\phi \left( {t,t_{0} } \right) = \phi_{0} \beta_{c} \left( {t - t_{0} } \right)$; $\phi_{0} = \phi_{RH} \beta \left( {f_{cm} } \right)\beta \left( {t_{0} } \right)$; $\phi_{RH} = 1 + \frac{{1 - RH/RH_{0} }}{{0.46\left( {h/h_{0} } \right)^{\frac{1}{3}} }}$; $\beta \left( {f_{cm} } \right) = \frac{5.3}{{\left( {f_{cm} /f_{cm0} } \right)^{0.5} }}$; $\beta \left( {t_{0} } \right) = \frac{1}{{0.1 + \left( {t_{0} /t_{1} } \right)^{0.2} }}$; $\beta_{c} \left( {t - t_{0} } \right) = \left[ {\frac{{\left( {t - t_{0} } \right)/t_{1} }}{{\beta_{H} + \left( {t - t_{0} } \right)/t_{1} }}} \right]^{0.3}$; $\beta_{H} = 150\left[ {1 + \left( {1.2\frac{RH}{{RH_{0} }}} \right)^{18} } \right]\frac{h}{{h_{0} }} + 250 \le 1500$; $\varphi_{0}$—nominal creep coefficient; $f_{cm}$—average cubic compressive strength of concrete at age 28 days, $f_{cm} = 0.8f_{cu,k} + 8$; $f_{cu,k}$—standard value of concrete cube compressive strength with 95% guarantee rate at age 28 days; $\beta_{c} \left( {t - t_{0} } \right)$—coefficient of development of the creep with time after loading; $h$—theoretical thickness of the member, where $h = 2A/u$; $A$—member cross-sectional area; $u$—perimeter of the contact surface between the component and the atmosphere; $RH$ —annual average relative humidity of the environment; $RH_{0}$ = 100%; $h_{0}$ = 100 mm; $t_{1}$ = 1 day; $f_{cmo}$ = 10 Mpa; $y_{si}$ and $y_{si} \left( t \right)$—respectively the vertical distance from the i-th layer of steel bars in the slab to the center of gravity of the slab converted section at time $t_{0}$ and time $t$; $\overline{y}$—vertical distance between the center of gravity of the beam and the slab at the moment of $t_{0}$; $\varepsilon_{o}$—initial strain at the center of gravity of the plate at the moment of $t_{0}$; $\varphi$—initial curvature of the composite beam at time $t_{0}$; $E_{s}$ and $E_{c}$—initial elastic moduli at time $t_{0}$ of the beam and plate, respectively; $A_{s}$ and $A_{c}$—cross-sectional areas at $t_{0}$ of the initial beam and plate sections, respectively; $I_{s}$ and $I_{c}$—moments of inertia at $t_{0}$ of the initial section of beam and plate, respectively; $E_{si}$—the elastic modulus of the i-the layer of steel bars in the slab; $A_{si}$—cross-sectional area of the i-th layer of steel bars in the slab; $n$—number of layers of reinforcement in the slab.

Since the internal and external loads do not change during the *t*_0_ − *t* time period, combined with the force on the section, the differential equation of the slip increment can be obtained.

3 $$ \frac{{{\text{d}}^{2} \Delta s\left( t \right)}}{{{\text{d}}x^{2} }} - \lambda^{2} \Delta \mathop s\limits^{{}} \left( t \right) = \mu_{1} \frac{{{\text{d}}M}}{{{\text{d}}x}} + \mu_{2} \frac{{{\text{d}}N}}{{{\text{d}}x}} $$

The differential equation of the interface shear force increment can then be obtained according to the relationship between the interface shear force and the slip.

4 $$ \frac{{{\text{d}}^{2} \Delta \tau_{s} \left( t \right)}}{{{\text{d}}x^{2} }} - \lambda^{2} \tau_{s} \left( t \right) = \mu_{s1} \frac{{{\text{d}}M}}{{{\text{d}}x}} + \mu_{s2} \frac{{{\text{d}}N}}{{{\text{d}}x}} $$

In the formula, $\lambda^{2} = \frac{{k_{L} y\left( t \right)^{2} }}{{E\left( {t,t_{0} } \right)I\left( t \right)}} + \frac{{k_{L} }}{{E\left( {t,t_{0} } \right)A\left( t \right)}}$; $\mu_{1} = - \frac{{y\left( t \right)\left[ {\gamma E_{c} \left( {t,t_{0} } \right)I_{c} \left( t \right) - \alpha_{1} } \right]}}{{E\left( {t,t_{0} } \right)I\left( t \right)}} - \frac{{\alpha_{1} }}{{E_{c} \left( {t,t_{0} } \right)A_{c} \left( t \right)}}$; $\mu_{2} = \delta + \frac{{y\left( t \right)\left[ {\gamma E_{c} \left( {t,t_{0} } \right)I_{c} \left( t \right) - \beta_{2} } \right]}}{{E\left( {t,t_{0} } \right)I\left( t \right)}} + \frac{{\beta_{1} }}{{E_{c} \left( {t,t_{0} } \right)A_{c} \left( t \right)}}$; $\mu_{s1} = - \frac{{k_{L} y\left( t \right)\left[ {\gamma E_{c} \left( {t,t_{0} } \right)I_{c} \left( t \right) - \alpha_{1} } \right]}}{{E\left( {t,t_{0} } \right)I\left( t \right)}} - \frac{{k_{L} \alpha_{1} }}{{E_{c} \left( {t,t_{0} } \right)A_{c} \left( t \right)}}$; $\mu_{s2} = k_{L} \delta + \frac{{k_{L} y\left( t \right)\left[ {\gamma E_{c} \left( {t,t_{0} } \right)I_{c} \left( t \right) - \beta_{2} } \right]}}{{E\left( {t,t_{0} } \right)I\left( t \right)}} + \frac{{k_{L} \beta_{1} }}{{E_{c} \left( {t,t_{0} } \right)A_{c} \left( t \right)}}$; $\gamma = \frac{{\phi \left( {t,t_{0} } \right)}}{EI}$; $\delta = \frac{{\phi \left( {t,t_{0} } \right)}}{{E_{c} A_{c} }}$; $\beta_{2} = \sum\nolimits_{i = 1}^{n} {E_{si} A_{si} y_{si} \left( t \right)\phi \left( {t,t_{0} } \right)\left[ {\frac{1}{{E_{c} A_{c} }} + \frac{{\overline{y}y_{si} }}{{E_{s} I_{s} + E_{c} I_{c} }}} \right]}$; $n$—the number of layers of steel bars in the plate; $k_{L}$—stiffness of the connecting piece, $k_{L} = k/m$; $k$—stiffness of a single connecting piece; $m$—the longitudinal spacing of the connecting piece.

The calculation formula of the interface slip increment under different loads can be obtained according to the boundary conditions.

##### Uniform load action

5 $$ \Delta s\left( t \right) = \frac{{q\left[ {2\left( {\alpha^{2} - \lambda^{2} } \right)\mu_{1} - 2\mu_{2} \beta } \right]}}{{2\left( {\alpha^{2} - \lambda^{2} } \right)\lambda^{3} \left( {e^{\lambda L/2} + e^{ - \lambda L/2} } \right)}}\left( {e^{ - \lambda x} - e^{\lambda x} } \right) - \frac{{\mu_{2} \beta q}}{{\left( {\alpha^{2} - \lambda^{2} } \right)\alpha^{3} \left( {e^{\alpha L/2} + e^{ - \alpha L/2} } \right)}}\left( {e^{\alpha x} - e^{ - \alpha x} } \right) + \frac{{\left( {\mu_{1} \alpha^{2} - \mu_{2} \beta } \right)qx}}{{\lambda^{2} \alpha^{2} }} $$

In the formula, $\alpha^{2} = \frac{{k_{L} }}{EA} + \frac{{k_{L} y^{2} }}{EI}$; $\beta = \frac{{k_{L} y}}{EI}$.

##### Symmetric concentrated load action

Bending segment:

6 $$ \Delta s\left( t \right) = - \left[ {\frac{{\mu_{1} }}{{2\lambda^{2} }} - \frac{{\mu_{2} \beta }}{{2\left( {\alpha^{2} - \lambda^{2} } \right)\lambda^{2} }}} \right]\frac{{P\left( {e^{{ - \lambda l_{0} /2}} + e^{{\lambda l_{0} /2}} } \right)}}{{e^{\lambda L} + 1}}\left( {e^{\lambda x} + e^{\lambda L - \lambda x} } \right) - \frac{{\mu_{2} \beta P\left( {e^{{ - \alpha l_{0} /2}} + e^{{\alpha l_{0} /2}} } \right)}}{{2\left( {\alpha^{2} - \lambda^{2} } \right)\alpha^{2} \left( {e^{\alpha L} + 1} \right)}}\left( {e^{\alpha L - \alpha x} + e^{\alpha x} } \right) + \frac{{\left( {\mu_{1} \alpha^{2} - \mu_{2} \beta } \right)P}}{{\lambda^{2} \alpha^{2} }} $$

Pure bend:

7 $$ \Delta s\left( t \right) = \left[ {\frac{{\mu_{1} }}{{2\lambda^{2} }} - \frac{{\mu_{2} \beta }}{{2\left( {\alpha^{2} - \lambda^{2} } \right)\lambda^{2} }}} \right]\frac{{P\left( {e^{{\lambda l_{0} /2}} - e^{{\lambda L - \lambda l_{0} /2}} } \right)}}{{e^{\lambda L} + 1}}\left( {e^{ - \lambda x} - e^{\lambda x} } \right) + \frac{{\mu_{2} \beta P\left( {e^{{\alpha L - \alpha l_{0} /2}} - e^{{\alpha l_{0} /2}} } \right)}}{{2\left( {\alpha^{2} - \lambda^{2} } \right)\alpha^{2} \left( {e^{\alpha L} + 1} \right)}}\left( {e^{\alpha x} - e^{ - \alpha x} } \right) $$

##### Arbitrary concentrated load action

To the left of the loading point:

8 $$ \Delta s\left( t \right) = \left[ {\frac{{\mu_{1} }}{{2\alpha_{t}^{2} }} - \frac{{\mu_{2} \beta }}{{2\left( {\alpha^{2} - \lambda^{2} } \right)\lambda^{2} }}} \right]\frac{{P\left( {e^{ - \lambda b} - e^{\lambda b - \lambda L} } \right)}}{{e^{\lambda L} - e^{ - \lambda L} }}\left( {e^{\lambda L + \lambda x} { + }e^{ - \lambda x} } \right) + \frac{{\mu_{2} \beta P\left( {e^{\alpha L - \alpha b} - e^{\alpha b} } \right)}}{{2\left( {\alpha^{2} - \lambda^{2} } \right)\alpha^{2} \left( {e^{2\alpha L} - 1} \right)}}\left( {e^{\alpha L + \alpha x} + e^{ - \alpha x} } \right) - \frac{{\left( {\mu_{1} \alpha^{2} - \mu_{2} \beta } \right)P\left( {L - 2b} \right)}}{{2\lambda^{2} \alpha^{2} L}} $$

To the right of the load point:

9 $$ \Delta s\left( t \right) = \left[ {\frac{{\mu_{1} }}{{2\lambda^{2} }} - \frac{{\mu_{2} \beta }}{{2\left( {\alpha^{2} - \lambda^{2} } \right)\lambda^{2} }}} \right]\frac{{P\left( {e^{ - \lambda b} - e^{\lambda b + \lambda L} } \right)}}{{e^{\lambda L} - e^{ - \lambda L} }}\left( {e^{\lambda x - \lambda L} { + }e^{ - \lambda x} } \right) + \frac{{\mu_{2} \beta P\left( {e^{ - \alpha b} - e^{\alpha L + \alpha b} } \right)}}{{2\left( {\alpha^{2} - \lambda^{2} } \right)\alpha^{2} \left( {e^{2\alpha L} - 1} \right)}}\left( {e^{\alpha L - \alpha x} + e^{\alpha x} } \right) - \frac{{\left( {\mu_{2} \beta - \mu_{1} \alpha^{2} } \right)P\left( {L + 2b} \right)}}{{2\lambda^{2} \alpha^{2} L}} $$

#### Energy variation method

Based on the energy variational method theory^44^, it is assumed that there is no slip between the steel beam and the CFRP sheet. The displacement of the steel beam is $U_{s1}$, the displacement of the concrete is $U_{c1}$, the deformation of the beam is $W_{1}$, and the displacement at the joint is $\Delta_{L1} = U_{s1} - U_{c1} { + }y\left( t \right)W_{1}^{{\prime}}$.

As such, the potential energies of the structural system at the *t* moment are shown below:

Strain energy of steel beam:

10 $$ \prod\nolimits_{s} = \frac{1}{2}E_{s} A_{s} \int {U_{s1}^{{\prime 2}} dx} + \frac{1}{2}E_{s} I_{s} \int {W_{1}^{{\prime\prime 2}} {\text{d}}x} $$

Strain energy of concrete:

11 $$ \begin{aligned} \prod\nolimits_{c} & = \frac{1}{2}\overline{E}_{c} \left( {t,t_{0} } \right)A_{c} \left( t \right)\int_{L} {U_{c1}^{{\prime 2}} } {\text{d}}x + \overline{E}_{c2} \left( {t,t_{0} } \right)A_{c} \left( t \right)\int_{L} {\left( {\delta NU_{c1}^{{\prime}} } \right){\text{d}}x}  + \frac{1}{2}\overline{E}_{c} \left( {t,t_{0} } \right)I_{c} \left( t \right)\int_{L} {W_{1}^{{\prime\prime 2}} } {\text{d}}x \\ & \quad + \overline{E}_{c} \left( {t,t_{0} } \right)I_{c} \left( t \right)\int_{L} {\left[ {\gamma M - \gamma y\left( {t_{0} } \right)N} \right]} W_{1}^{{\prime\prime}} {\text{d}}x + \int_{L} {\left( {\beta_{1} N - \alpha_{1} M} \right)U_{c1}^{{\prime}} {\text{d}}x} + \int_{L} {\left( {\beta_{2} N - \alpha_{2} M} \right)W_{1}^{{\prime\prime}} {\text{d}}x} \\ \end{aligned} $$

Strain energy at the joint:

12 $$ \prod\nolimits_{L1} { = \frac{1}{2}\int_{L} {k_{L} \left[ {U_{s1} - U_{c1} { + }y\left( t \right)W_{1}^{{\prime}} } \right]^{2} {\text{d}}x} } $$

The total potential energy increment of the beam is shown in Eq. (13):

13 $$ \begin{aligned} \prod  &  = \frac{1}{2}E_{s} A_{s} \int {U_{s1}^{{\prime 2}} {\text{d}}x} + \frac{1}{2}E_{s} I_{s} \int {W_{1}^{{\prime\prime 2}} {\text{d}}x}  { + }\frac{1}{2}\overline{E}_{c} \left( {t,t_{0} } \right)A_{c} \left( t \right)\int_{L} {U_{c1}^{{\prime}2} } {\text{d}}x + \overline{E}_{c2} \left( {t,t_{0} } \right)A_{c} \left( t \right)\int_{L} {\left( {\delta NU_{c1}^{{\prime}} } \right){\text{d}}x} \\ & \quad + \frac{1}{2}\overline{E}_{c} \left( {t,t_{0} } \right)I_{c} \left( t \right)\int_{L} {W_{1}^{{\prime\prime}2} } {\text{d}}x + \overline{E}_{c} \left( {t,t_{0} } \right)I_{c} \left( t \right)\int_{L} {\left[ {\gamma M - \gamma y\left( {t_{0} } \right)} \right]} W_{1}^{{\prime\prime}} {\text{d}}x + + \int_{L} {\left( {\beta_{1} N - \alpha_{1} M} \right)U_{c1}^{{\prime}} {\text{d}}x} \\ & \quad + \int_{L} {\left( {\beta_{2} N - \alpha_{2} M} \right)W_{1}^{{\prime\prime}} {\text{d}}x} + \frac{1}{2}\int_{L} {k_{L} \left[ {U_{s1} - U_{c1} { + }y\left( t \right)W_{1}^{{\prime}} } \right]^{2} {\text{d}}x} \\ \end{aligned} $$

According to the principle of minimum potential energy, the variation of Eq. (13) is further integrated step-by-step to obtain the following:

14 $$ \begin{aligned} \delta \prod  & = E_{s} A_{s} \int {U_{s1}^{{\prime}} \delta U_{s1}^{{\prime}} {\text{d}}x} + E_{s} I_{s} \int {W_{1}^{{\prime\prime}} \delta W_{1}^{{\prime\prime}} {\text{d}}x} + \left[ {\overline{E}_{c} \left( {t,t_{0} } \right)A_{c} U_{c1}^{{\prime}} + \overline{E}_{c} \left( {t,t_{0} } \right)A_{c} \delta N - \alpha_{1} M + \beta_{1} N} \right]\delta U_{c1}^{{\prime}} {\text{d}}x \\ & \quad + \left\{ {\beta_{2} N - \alpha_{2} M} \right.\left. { + \overline{E}_{c} \left( {t,t_{0} } \right)I_{c} \left( t \right)\left[ {\gamma M - \gamma y\left( {t_{0} } \right)N} \right]} \right\}\delta W_{1}^{{\prime\prime}} {\text{d}}x \\ & \quad + k_{L} \left[ {U_{s1} - U_{c1} + y\left( t \right)W_{1}^{{\prime}} } \right]\left[ {\delta U_{s1} - \delta U_{c1} + y\left( t \right)\delta W_{1}^{{\prime}} } \right]{\text{d}}x \\ \end{aligned} $$

Furthermore, $\delta U_{s1}^{{}}$, $\delta U_{c1}^{{}}$, $\delta W$ are independent quantities, so Eq. (14) is integrated by division and reduced.

15 $$ \begin{aligned} & - E_{s} A_{s} U_{s}^{{\prime\prime}} + k_{L} \left[ {U_{s1} - U_{c1} + y\left( t \right)W_{1}^{{\prime}} } \right] = 0 \\ & \overline{E}_{c} \left( {t,t_{0} } \right)A_{c} \left( t \right)U_{c1}^{{\prime\prime}} + \left[ {\overline{E}_{c} \left( {t,t_{0} } \right)A_{c} \left( t \right)\delta + \beta_{1} } \right]\frac{{{\text{d}}N}}{{{\text{d}}x}} - \alpha_{1} \frac{{{\text{d}}M}}{{{\text{d}}x}} + k_{L} \left[ {U_{s1} - U_{c1} + y\left( t \right)W_{1}^{{\prime}} } \right] = 0 \\ & \left[ {E_{s} I_{s} + \overline{E}_{c} \left( {t,t_{0} } \right)I_{c} \left( t \right)} \right]W_{{}}^{(4)} \left[ {\delta \overline{E}_{c} \left( {t,t_{0} } \right)I_{c} \left( t \right) - \alpha_{2} } \right]\frac{{{\text{d}}^{2} M}}{{{\text{d}}x^{2} }} \\ & \left[ {E_{s} I_{s} + \overline{E}_{c} \left( {t,t_{0} } \right)I_{c} \left( t \right)} \right]W_{{}}^{(4)} \left[ {\delta \overline{E}_{c} \left( {t,t_{0} } \right)I_{c} \left( t \right) - \alpha_{2} } \right]\frac{{{\text{d}}^{2} M}}{{{\text{d}}x^{2} }} \\ \end{aligned} $$

According to the balance of internal forces, the governing differential equation of the slip can then be obtained.

16 $$ \frac{{{\text{d}}^{2} \Delta s\left( t \right)}}{{{\text{d}}x^{2} }} - \lambda^{2} \Delta \mathop s\limits^{{}} \left( t \right) = \mu_{1} \frac{{{\text{d}}M}}{{{\text{d}}x}} + \mu_{2} \frac{{{\text{d}}N}}{{{\text{d}}x}} $$

### Design parameter analysis

#### Load

**Figure 2:**
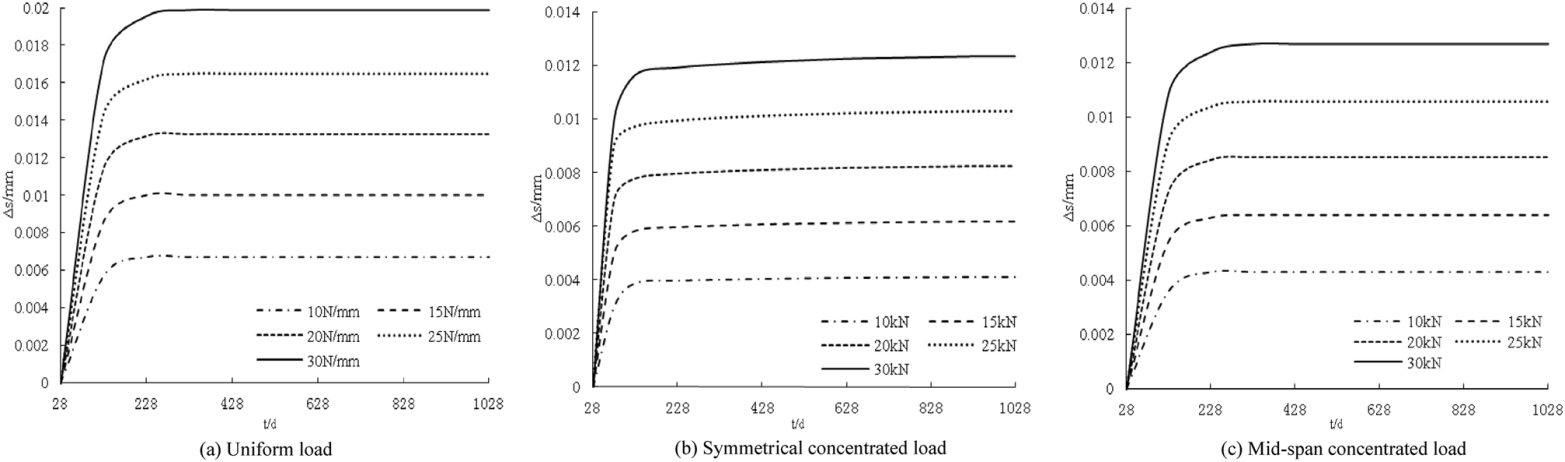
Influence of loading under creep on interface slip increment concentrated load.

The distribution curve of the slip increment with age under different loads is shown in Fig. 2. The interfacial slip increment between the steel beam and the concrete slab shows a nonlinear distribution with age. The interface slip increments at 28 days are all zero. With the increase in age, the slip increments gradually increase. The increase is larger within 100 days, and the slip increments are basically unchanged from 100 to 1028 days. The interface slip increment increases with increasing load. The greater the load, the steeper the slip increment curve. When the load increases by 5 N/mm (5 kN), the slip increment increases by approximately 0.004 mm, 0.002 mm, and 0.002 mm.

#### Connection stiffness

**Figure 3:**
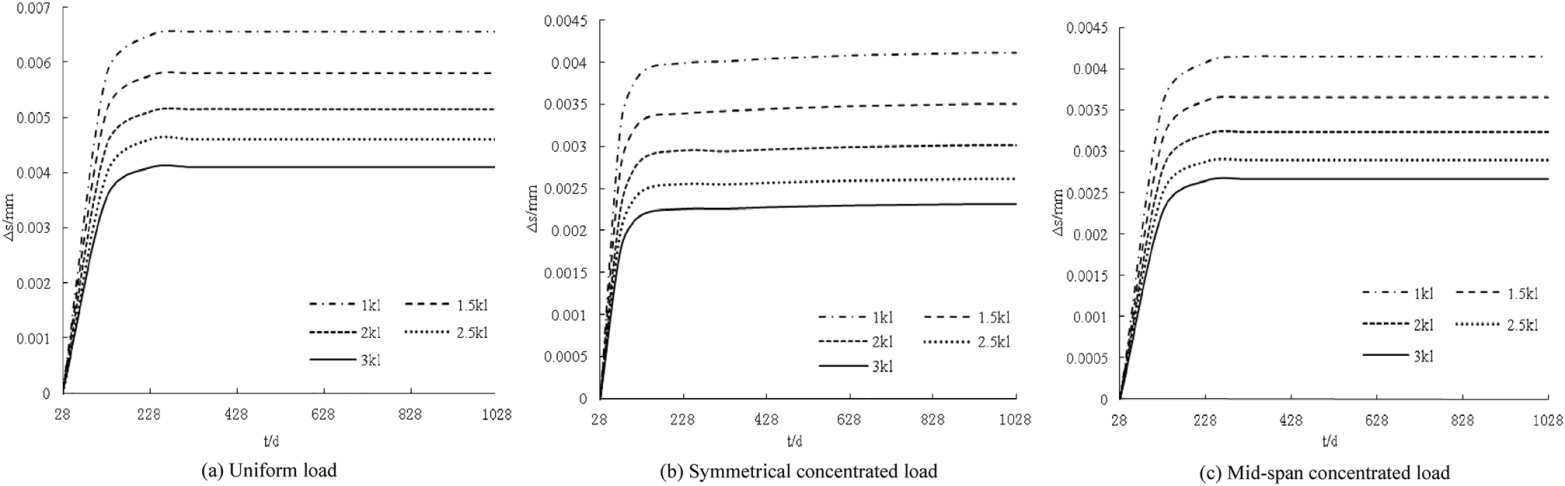
Influence of coupling stiffness under creep on interface slip increment.

The distribution curves of the slip increments with age under different stiffnesses are shown in Fig. 3. The interface slip increment decreases with increasing stiffness. The greater the stiffness, the smoother the slip increment curve. The amount of change to the interfacial slip increment decreases gradually with each increase in stiffness.

## Incremental analysis of axial force

### Incremental calculation of axial force

The differential equation of the axial force increment can be obtained from the relationship $\frac{{{\text{d}}\Delta N\left( t \right)}}{{{\text{d}}x}} = \Delta \tau_{s} \left( t \right) = k_{L} \Delta s\left( t \right)$ between the axial force increment and the slip increment.

17 $$ \frac{{{\text{d}}^{2} \Delta N\left( t \right)}}{{{\text{d}}x^{2} }} - \lambda^{2} \Delta N\left( t \right) = \mu_{1} k_{L} M + \mu_{2} k_{L} N $$

where $$\lambda^{2} = \frac{{k_{L} }}{{E\left( {t,t_{0} } \right)A\left( t \right)}} + \frac{{k_{L} y^{2} \left( t \right)}}{{E\left( {t,t_{0} } \right)I\left( t \right)}}$$; $$\mu_{1} = - \frac{{y\left( t \right)\left[ {\gamma E_{c} \left( {t,t_{0} } \right)I_{c} \left( t \right) - \alpha_{2} } \right]}}{{E\left( {t,t_{0} } \right)I\left( t \right)}} - \frac{{\alpha_{1} }}{{E_{c} \left( {t,t_{0} } \right)A_{c} \left( t \right)}}$$; $$\mu_{2} = \delta + \frac{{y\left( t \right)\left[ {\gamma y\left( {t_{0} } \right)E_{c} \left( {t,t_{0} } \right)I_{c} \left( t \right) - \beta_{2} } \right]}}{{E\left( {t,t_{0} } \right)I\left( t \right)}} + \frac{{\beta_{1} }}{{E_{c} \left( {t,t_{0} } \right)A_{c} \left( t \right)}}$$.

The calculation formula of the axial force increment under different loads can be obtained according to the boundary conditions.

#### Uniform load action

18 $$ \begin{aligned} \Delta N\left( t \right) & = - \frac{{k_{L} q}}{{e^{ - \lambda L/2} + e^{\lambda L/2} }}\left[ {\frac{{\mu_{1} }}{{\lambda^{4} }} - \frac{{\mu_{2} \beta }}{{\left( {\alpha^{2} - \lambda^{2} } \right)\lambda^{4} }}} \right]\left( {e^{\lambda x} + e^{ - \lambda x} } \right) - \frac{{\mu_{2} \beta k_{L} q}}{{\left( {\alpha^{2} - \lambda^{2} } \right)\alpha^{4} \left( {e^{\alpha L/2} + e^{ - \alpha L/2} } \right)}}\left( {e^{\alpha x} + e^{ - \alpha x} } \right) \\ & \quad + \left( {\frac{{\mu_{1} k_{L} q}}{{2\lambda^{2} }} - \frac{{\mu_{2} \beta k_{L} q}}{{2\alpha^{2} \lambda^{2} }}} \right)x^{2} + \frac{{\mu_{1} k_{L} q}}{{{8}\lambda^{4} }}\left( {{8} - L^{2} \lambda^{2} } \right) - \frac{{\mu_{2} \beta k_{L} q}}{{8\alpha^{4} \lambda^{4} }}\left( {8\alpha^{2} { + }8\lambda^{2} - L^{2} \lambda^{2} \alpha^{2} } \right) \\ \end{aligned} $$

#### Symmetric concentrated load action

Bending segment:

19 $$ \Delta N\left( t \right) = \left[ {\frac{{\mu_{1} }}{{2\lambda^{3} }} - \frac{{\mu_{2} \beta }}{{2\left( {\alpha^{2} - \lambda^{2} } \right)\lambda^{3} }}} \right]\frac{{k_{L} P\left( {e^{{ - \lambda l_{0} /2}} + e^{{\lambda l_{0} /2}} } \right)}}{{e^{\lambda L} + 1}}\left( {e^{\lambda L - \lambda x} - e^{\lambda x} } \right) + \frac{{k_{L} P\left( {\mu_{1} \alpha^{2} - \mu_{2} \beta } \right)}}{{2\lambda^{2} \alpha^{2} }}\left( {2x - L} \right) + \frac{{\mu_{2} \beta k_{L} P\left( {e^{{ - \alpha l_{0} /2}} + e^{{\alpha l_{0} /2}} } \right)}}{{2\left( {\alpha^{2} - \lambda^{2} } \right)\alpha^{3} \left( {e^{\alpha L} + 1} \right)}}\left( {e^{\alpha L - \alpha x} - e^{\alpha x} } \right) $$

Pure bend:

20 $$ \Delta N\left( t \right) = \left[ {\frac{{\mu_{1} }}{{2\lambda^{3} }} - \frac{{\mu_{2} \beta }}{{2\left( {\alpha^{2} - \lambda^{2} } \right)\lambda^{3} }}} \right]\frac{{k_{L} P\left( {e^{{\lambda L - \lambda l_{0} /2}} - e^{{\lambda l_{0} /2}} } \right)}}{{e^{\lambda L} + 1}}\left( {e^{\lambda x} + e^{ - \lambda x} } \right) + \frac{{\mu_{2} \beta k_{L} P\left( {e^{{\alpha L - \alpha l_{0} /2}} - e^{{\alpha l_{0} /2}} } \right)}}{{2\left( {\alpha^{2} - \lambda^{2} } \right)\alpha^{3} \left( {e^{\alpha L} + 1} \right)}}\left( {e^{\alpha x} + e^{ - \alpha x} } \right) + \frac{{\left( {\mu_{2} \beta - \mu_{1} \alpha^{2} } \right)k_{L} P\left( {L - l_{0} } \right)}}{{2\lambda^{2} \alpha^{2} }} $$

#### Arbitrary concentrated load action

To the left of the loading point:

21 $$ \begin{aligned} \Delta N\left( t \right) & = \frac{{\mu_{2} \beta k_{L} P\left( {e^{\alpha L - \alpha b} - e^{\alpha b} } \right)}}{{2\left( {\alpha^{2} - \lambda^{2} } \right)\alpha^{3} \left( {e^{2\alpha L} - 1} \right)}}\left( {e^{\alpha L + \alpha x} - e^{ - \alpha x} } \right) - \frac{{\left( {\mu_{1} \alpha^{2} - \mu_{2} \beta } \right)k_{L} P\left( {L - 2b} \right)}}{{4\lambda^{2} \alpha^{2} L}}\left( {2x + L} \right) \\ & \quad + \left[ {\frac{{\mu_{1} }}{{2\lambda^{3} }} - \frac{{\mu_{2} \beta }}{{2\left( {\alpha^{2} - \lambda^{2} } \right)\lambda^{3} }}} \right]\frac{{k_{L} P\left[ {\left( {e^{\lambda L - \lambda b} - e^{\lambda b} } \right)e^{\lambda x} + \left( {e^{\lambda b - \lambda L} - e^{ - \lambda b} } \right)e^{ - \lambda x} } \right]}}{{e^{\lambda L} - e^{ - \lambda L} }} \\ \end{aligned} $$

To the right of the load point:

22 $$ \begin{aligned} \Delta N\left( t \right) & = \frac{{\mu_{2} \beta k_{L} P\left( {e^{\alpha L + \alpha b} - e^{ - \alpha b} } \right)}}{{2\left( {\alpha^{2} - \lambda^{2} } \right)\alpha^{3} \left( {e^{2\alpha L} - 1} \right)}}\left( {e^{\alpha L - \alpha x} - e^{\alpha x} } \right) + \frac{{\left( {\mu_{1} \alpha^{2} - \mu_{2} \beta } \right)k_{L} P\left( {L + 2b} \right)}}{{4\lambda^{2} \alpha^{2} L}}\left( {2x - L} \right) \\ & \quad + \left[ {\frac{{\mu_{1} }}{{2\lambda^{3} }} - \frac{{\mu_{2} \beta }}{{2\left( {\alpha^{2} - \lambda^{2} } \right)\lambda^{3} }}} \right]\frac{{k_{L} P\left[ {\left( {e^{ - \lambda b - \lambda L} - e^{\lambda b} } \right)e^{\lambda x} - \left( {e^{ - \lambda b} - e^{\lambda b + \lambda L} } \right)e^{ - \lambda x} } \right]}}{{e^{\lambda L} - e^{ - \lambda L} }} \\ \end{aligned} $$

### Analysis of design parameters

#### Load

**Figure 4:**
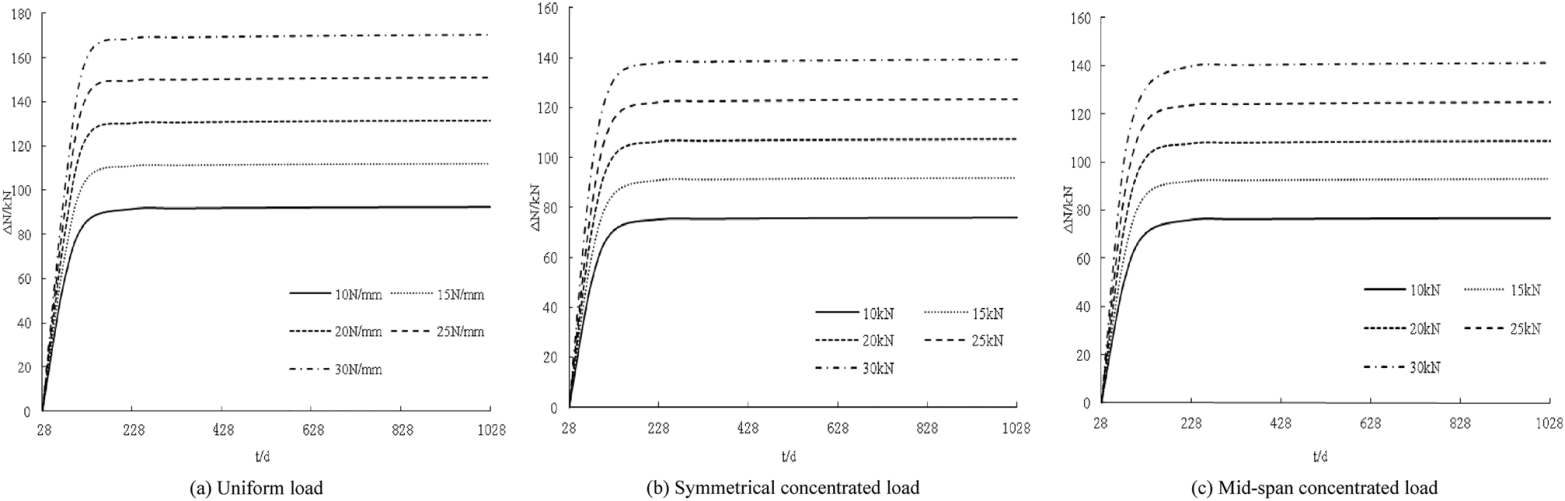
Influence of loading under creep on axial force increment.

The distribution curve of the axial force increment with age under different loads is shown in Fig. 4. The axial force increments at 28 days are all zero. With increasing age, the axial force increment gradually increases within 100 days. From 100 to 1028 days, the increase is larger and the axial force increment is basically unchanged; the axial force increment increases with increasing load, and the larger the load, the steeper the axial force increment curve. Every 5 N/mm (5 kN), the axial force increments increase by approximately 19.4 kN, 15.9 kN, and 16.1 kN.

#### Connection stiffness

**Figure 5:**
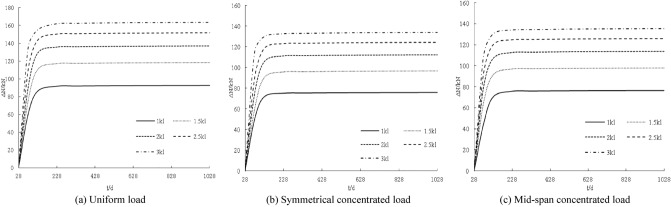
Coupling influence of stiffness under creep on axial force increment.

The distribution curve of the axial force increment with age under different stiffness values is shown in Fig. 5. The axial force increment increases with increasing connection stiffness. The greater the load, the steeper the change in the curve of the axial force increment. Similarly, the greater the increase in the connection stiffness, the smaller the increase in the axial force increment.

## Deformation incremental analysis of composite beams

### Deformation increment calculation of composite beam

According to the relationship between deformation and curvature, combined with the differential equation of the slip increment, the differential equation of the deformation increment can be obtained.

23 $$ \frac{{{\text{d}}^{4} \Delta W\left( t \right)}}{{{\text{d}}x^{4} }} - \lambda^{2} \frac{{{\text{d}}^{2} \Delta W\left( t \right)}}{{{\text{d}}x^{2} }} = \mu_{1}^{{\prime}} M + \mu_{2}^{{\prime}} N + \mu_{3}^{{\prime}} \frac{{{\text{d}}^{2} M}}{{{\text{d}}x^{2} }} + \mu_{4}^{{\prime}} \frac{{{\text{d}}^{2} N}}{{{\text{d}}x^{2} }} $$

where, $$\lambda^{2} = \frac{{k_{L} }}{{E\left( {t,t_{0} } \right)A\left( t \right)}} + \frac{{k_{L} y\left( t \right)^{2} }}{{E\left( {t,t_{0} } \right)I\left( t \right)}}$$; $$\mu_{1}^{{}} = \frac{{k_{L} }}{{E^{2} \left( {t,t_{0} } \right)I\left( t \right)A\left( t \right)}}\left[ {E_{c} \left( {t,t_{0} } \right)I_{c} \left( t \right)\gamma - \alpha_{2} } \right] - \frac{{k_{L} y\left( t \right)}}{{E\left( {t,t_{0} } \right)I\left( t \right)}}\frac{{\alpha_{1} }}{{E_{c} \left( {t,t_{0} } \right)A_{c} \left( t \right)}}$$; $$\mu_{2}^{{}} = - \frac{{k_{L} }}{{E^{2} \left( {t,t_{0} } \right)I\left( t \right)A\left( t \right)}}\left[ {E_{c} \left( {t,t_{0} } \right)I_{c} \left( t \right)\gamma y - \beta_{2} } \right] + \frac{{y\left( t \right)k_{L} }}{{E\left( {t,t_{0} } \right)I\left( t \right)}}\left[ {\delta + \frac{{\beta_{1} }}{{E_{c} \left( {t,t_{0} } \right)A_{c} \left( t \right)}}} \right]$$;$$\mu_{3}^{{}} = - \frac{{E_{c} \left( {t,t_{0} } \right)I_{c} \left( t \right)\gamma - \alpha_{2} }}{{E\left( {t,t_{0} } \right)I\left( t \right)}}$$; $$\mu_{4}^{{}} = \frac{{E_{c} \left( {t,t_{0} } \right)I_{c} \left( t \right)\gamma y - \beta_{2} }}{{E\left( {t,t_{0} } \right)I\left( t \right)}}$$.

According to the boundary conditions, the calculation formula of the deformation increment under different loads can be obtained.

#### Uniform load action

24 $$ \begin{aligned} \Delta W\left( t \right) & = \left( {e^{\lambda x} + e^{ - \lambda x} } \right)\left\{ {\frac{q}{{\left( {1 + e^{ - \lambda L} } \right)\lambda^{4} }}\left[ { - \frac{{\mu_{1}^{{}} e^{ - \lambda L/2} }}{{\lambda^{2} }} + \frac{{\mu_{2}^{{}} \beta e^{ - \lambda L/2} }}{{\left( {\alpha^{2} - \lambda^{2} } \right)\lambda^{2} }} - \mu_{3}^{{}} e^{ - \lambda L/2} } \right.\left. { + \frac{{\mu_{4}^{{}} \beta e^{ - \lambda L/2} }}{{\alpha^{2} - \lambda^{2} }}} \right]} \right\} - \left( {\frac{{\mu_{2}^{{}} \beta q}}{{24\lambda^{2} \alpha^{2} }} - \frac{{\mu_{1}^{{}} q}}{{24\lambda^{2} }}} \right)x^{4} \\ & \quad - \left( {\frac{{\mu_{1}^{{}} qL^{2} }}{{16\lambda^{2} }} - \frac{{\mu_{1}^{{}} q}}{{2\lambda^{4} }} + \frac{{\mu_{2}^{{}} \beta q}}{{2\lambda^{2} \alpha^{4} }} + \frac{{\mu_{2}^{{}} \beta q}}{{2\lambda^{4} \alpha^{2} }} - \frac{{\mu_{2}^{{}} \beta qL^{2} }}{{16\lambda^{2} \alpha^{2} }} - \frac{{\mu_{3}^{{}} q}}{{2\lambda^{2} }} + \frac{{\mu_{4}^{{}} \beta q}}{{2\lambda^{2} \alpha^{2} }}} \right)x^{2} - \frac{{\left( {\mu_{2}^{{}} + \mu_{4}^{{}} \alpha^{2} } \right)\beta qe^{\alpha L/2} }}{{\left( {\alpha^{2} - \lambda^{2} } \right)\alpha^{6} \left( {e^{\alpha L} + 1} \right)}}\left( {e^{ - \alpha x} + e^{\alpha x} } \right) \\ & \quad + \mu_{4}^{{}} \beta q\left[ {\frac{{L^{2} }}{{8\lambda^{2} \alpha^{2} }} - \frac{1}{{\left( {\alpha^{2} - \lambda^{2} } \right)\lambda^{4} }} + \frac{1}{{\left( {\alpha^{2} - \lambda^{2} } \right)\alpha^{4} }}} \right] + \frac{{\mu_{1}^{{}} q}}{{\lambda^{6} }}\left( {1 + \frac{{5\lambda^{4} L^{4} }}{384} - \frac{{L^{2} \lambda^{2} }}{8}} \right) - \frac{{\mu_{3}^{{}} q}}{{8\lambda^{4} }}\left( {\lambda^{2} L^{2} - 8} \right) \\ & \quad + \mu_{2}^{{}} \beta q\left[ {\frac{{L^{2} }}{{8\lambda^{2} \alpha^{4} }} + \frac{{L^{2} }}{{8\lambda^{4} \alpha^{2} }} - \frac{{L^{4} }}{{384\lambda^{2} \alpha^{2} }} - \frac{1}{{\left( {\alpha^{2} - \lambda^{2} } \right)\lambda^{6} }} + \frac{1}{{\left( {\alpha^{2} - \lambda^{2} } \right)\alpha^{6} }}} \right] \\ \end{aligned} $$

#### Symmetric concentrated load action

Bending segment:

25 $$ \begin{aligned} \Delta W\left( t \right) & = - P\left\{ {\frac{{\mu_{1}^{{}} \left( {e^{{\lambda l_{0} /2}} + e^{{ - \lambda l_{0} /2}} } \right)}}{{2\lambda^{5} \left( {1 + e^{\lambda L} } \right)}} - \frac{{\mu_{2}^{{}} \beta \left( {e^{{\lambda l_{0} /2}} + e^{{ - \lambda l_{0} /2}} } \right)}}{{2\left( {\alpha^{2} - \lambda^{2} } \right)\lambda^{5} \left( {1 + e^{\lambda L} } \right)}}} \right. + \frac{{\mu_{3}^{{}} \left( {e^{{\lambda l_{0} /2}} + e^{{ - \lambda l_{0} /2}} } \right)}}{{2\lambda^{3} \left( {1 + e^{{\lambda_{t} L}} } \right)}}\left. { - \frac{{\mu_{4}^{{}} \beta \left( {e^{{\lambda l_{0} /2}} + e^{{ - \lambda l_{0} /2}} } \right)}}{{2\left( {\alpha^{2} - \lambda^{2} } \right)\lambda^{3} \left( {1 + e^{\lambda L} } \right)}}} \right\}\left( {e^{\lambda x} + e^{\lambda L} e^{ - \lambda x} } \right) \\ & \quad + \frac{{\left( {\mu_{2}^{{}} \beta - \mu_{1}^{{}} \alpha^{2} } \right)P}}{{12\lambda^{2} \alpha^{2} }}\left( {3Lx^{2} - 2x^{3} } \right) - \left\{ {\frac{{\mu_{4}^{{}} \beta P}}{{\alpha^{2} \lambda^{2} }} + \frac{{\mu_{3}^{{}} P}}{{\lambda^{2} }} - \mu_{2}^{{}} \beta P\left[ {\frac{{\lambda^{2} + \alpha^{2} }}{{\lambda^{4} \alpha^{4} }} + \frac{{l_{0}^{2} }}{{8\lambda^{2} \alpha^{2} }}} \right] + \frac{{\mu_{1}^{{}} P}}{{\lambda^{4} }}\left( {1 + \frac{{\lambda^{2} l_{0}^{2} }}{8}} \right)} \right\}x + \frac{{\mu_{4}^{{}} \beta PL}}{{2\alpha^{2} \lambda^{2} }} - \frac{{\mu_{3}^{{}} PL}}{{2\lambda^{2} }} \\ & \quad - \frac{{\mu_{1}^{{}} PL}}{{2\lambda^{4} }}\left( {1 + \frac{{\lambda^{2} l_{0}^{2} }}{8} - \frac{{\lambda^{2} L^{2} }}{12}} \right) + \frac{{\mu_{2}^{{}} \beta PL}}{2}\left[ {\frac{{\lambda^{2} + \alpha^{2} }}{{\lambda^{4} \alpha^{4} }} + \frac{{3l_{0}^{2} - 2L^{2} }}{{24\lambda^{2} \alpha^{2} }}} \right] + \frac{{\left( {\mu_{2}^{{}} + \mu_{4}^{{}} \alpha^{2} } \right)\beta P\left( {e^{{ - \alpha l_{0} /2}} + e^{{\alpha l_{0} /2}} } \right)}}{{2\left( {\alpha^{2} - \lambda^{2} } \right)\alpha^{5} \left( {e^{\alpha L} + 1} \right)}}\left( {e^{\alpha L - \alpha x} - e^{\alpha x} } \right) \\ \end{aligned} $$

Pure bend:

26 $$ \begin{aligned} \Delta W\left( t \right) & = \left[ {\frac{{P\mu_{1}^{{}} \left( {e^{{ - \lambda l_{0} /2}} - e^{{\lambda l_{0} /2 - \lambda L}} } \right)}}{{2\lambda^{5} \left( {1 + e^{ - \lambda L} } \right)}} + \frac{{P\mu_{2}^{{}} \beta \left( {e^{{\lambda l_{0} /2 - \lambda L}} - e^{{ - \lambda l_{0} /2}} } \right)}}{{2\left( {\alpha^{2} - \lambda^{2} } \right)\lambda^{5} \left( {1 + e^{ - \lambda L} } \right)}} + \frac{{P\mu_{4}^{{}} \beta \left( {e^{{\lambda l_{0} /2 - \lambda L}} - e^{{ - \lambda l_{0} /2}} } \right)}}{{2\left( {\alpha^{2} - \lambda^{2} } \right)\lambda^{3} \left( {1 + e^{ - \lambda L} } \right)}} - \frac{{P\mu_{3}^{{}} \left( {e^{{\lambda l_{0} /2 - \lambda L}} - e^{{ - \lambda l_{0} /2}} } \right)}}{{2\lambda^{3} \left( {1 + e^{ - \lambda L} } \right)}}} \right]\left( {e^{\lambda x} + e^{ - \lambda x} } \right) \\ & \quad - \frac{{\mu_{1}^{{}} P\left( {L - l_{0} } \right)}}{{4\lambda^{2} }}x^{2} + \frac{{\mu_{2}^{{}} \beta P\left( {L - l_{0} } \right)}}{{4\lambda^{2} \alpha^{2} }}x^{2} + \frac{{\left( {\mu_{2}^{{}} + \mu_{4}^{{}} \alpha^{2} } \right)\beta P\left( {e^{{\alpha L - \alpha l_{0} /2}} - e^{{\alpha l_{0} /2}} } \right)}}{{2\left( {\alpha^{2} - \lambda^{2} } \right)\alpha^{5} \left( {e^{\alpha L} + 1} \right)}}\left( {e^{\alpha x} + e^{ - \alpha x} } \right) + \frac{{\mu_{4}^{{}} \beta P\left( {L - l_{0} } \right)}}{{2\alpha^{2} \lambda^{2} }} - \frac{{\mu_{3}^{{}} P\left( {L - l_{0} } \right)}}{{2\lambda^{2} }} \\ & \quad + \frac{{\mu_{2}^{{\prime}} \beta P}}{2}\left[ {\frac{{\left( {\lambda^{2} + \alpha^{2} } \right)\left( {L - l_{0} } \right)}}{{\lambda^{4} \alpha^{4} }} + \frac{{3Ll_{0}^{2} - 2L^{3} - l_{0}^{3} }}{{24\lambda^{2} \alpha^{2} }}} \right] + \frac{{\mu_{1}^{{}} P}}{{2\lambda^{4} }}\left( {l_{0} - L + \frac{{\lambda^{2} l_{0}^{3} }}{24} - \frac{{\lambda^{2} Ll_{0}^{2} }}{8} + \frac{{\lambda^{2} L^{3} }}{12}} \right) \\ \end{aligned} $$

#### Arbitrary concentrated load action

To the left of the loading point:

27 $$ \begin{aligned} \Delta W\left( t \right) & = P\left\{ {\frac{{\mu_{1}^{{}} \left( {e^{\lambda b - \lambda L} - e^{ - \lambda b} } \right)}}{{2\lambda^{5} \left( {1 - e^{ - 2\lambda L} } \right)}} + \frac{{\mu_{2}^{{}} \beta \left( {e^{ - \lambda b} - e^{\lambda b - \lambda L} } \right)}}{{2\left( {\alpha^{2} - \lambda^{2} } \right)\lambda^{5} \left( {1 - e^{ - 2\lambda L} } \right)}}} \right. + \frac{{\mu_{3}^{{}} \left( {e^{\lambda b - \lambda L} - e^{ - \lambda b} } \right)}}{{2\lambda^{3} \left( {1 - e^{ - 2\lambda L} } \right)}}\left. { + \frac{{\mu_{4}^{{}} \beta \left( {e^{ - \lambda b} - e^{\lambda b - \lambda L} } \right)}}{{2\lambda^{3} \left( {\alpha^{2} - \lambda^{2} } \right)\left( {1 - e^{ - 2\lambda L} } \right)}}} \right\}\left( {e^{ - \lambda L} e^{ - \lambda x} - e^{\lambda x} } \right) \\ & \quad - \frac{{\mu_{1}^{{}} P\left( {L - 2b} \right)}}{{24\lambda^{2} L}}\left( {3Lx^{2} + 2x^{3} } \right) + \frac{{\mu_{2}^{{}} \beta P\left( {e^{\alpha L - \alpha b} - e^{\alpha b} } \right)}}{{2\left( {\alpha^{2} - \lambda^{2} } \right)\alpha^{5} \left( {e^{2\alpha L} - 1} \right)}}\left( {e^{\alpha L + \alpha x} - e^{ - \alpha x} } \right) + \frac{{\mu_{4}^{{}} \beta P\left( {e^{\alpha L - \alpha b} - e^{\alpha b} } \right)}}{{2\left( {\alpha^{2} - \lambda^{2} } \right)\alpha^{3} \left( {e^{2\alpha L} - 1} \right)}}\left( {e^{\alpha L + \alpha x} - e^{ - \alpha x} } \right) + \frac{{\mu_{2}^{{}} \beta P\left( {L - 2b} \right)}}{{24\lambda^{2} \alpha^{2} L}}\left( {2x^{3} + 3Lx^{2} } \right) \\ & \quad + \left[ { - \frac{{\mu_{2}^{{}} \beta P}}{{\lambda^{2} \alpha^{2} L}}\left( {\frac{{bL^{2} }}{12} + \frac{{b^{3} }}{6} - \frac{{Lb^{2} }}{4}} \right) + \frac{{\mu_{1}^{{}} P}}{{\lambda^{2} L}}\left( {\frac{b}{{\lambda^{2} }} - \frac{L}{{2\lambda^{2} }} + \frac{{bL^{2} }}{12} + \frac{{b^{3} }}{6} - \frac{{b^{2} L}}{4}} \right) + \frac{{\left( {\alpha^{2} + \lambda^{2} } \right)\mu_{2}^{{}} \beta P\left( {L - 2b} \right)}}{{2\alpha^{4} \lambda^{4} L}} + \frac{{\left( {\mu_{3}^{{}} \alpha^{2} - \mu_{4}^{{}} \beta } \right)P}}{{2\lambda^{2} \alpha^{2} L}}\left( {2b - L} \right)} \right]x \\ & \quad + \frac{{\left( {\alpha^{2} + \lambda^{2} } \right)\mu_{2}^{{}} \beta P\left( {L - 2b} \right)L}}{{4\alpha^{4} \lambda^{4} L}} - \frac{{\mu_{2}^{{}} \beta P}}{{2\lambda^{2} \alpha^{2} }}\left( {\frac{{b^{3} }}{6} - \frac{{Lb^{2} }}{4} + \frac{{L^{3} }}{24}} \right) + \frac{{\mu_{1}^{{}} P}}{{2\lambda^{2} }}\left( {\frac{b}{{\lambda^{2} }} - \frac{L}{{2\lambda^{2} }} + \frac{{b^{3} }}{6} - \frac{{b^{2} L}}{4} + \frac{{L^{3} }}{24}} \right) + \frac{{\left( {\mu_{3}^{{}} \alpha^{2} - \mu_{4}^{{}} \beta } \right)PL}}{{4\lambda^{2} \alpha^{2} L}}\left( {2b - L} \right) \\ \end{aligned} $$

To the right of the load point:

28 $$ \begin{aligned} \Delta W\left( t \right) & = P\left\{ {\frac{{\mu_{1}^{{}} \left( {e^{\lambda b} - e^{ - \lambda b - \lambda L} } \right)}}{{2\lambda^{5} \left( {1 - e^{ - 2\lambda L} } \right)}} + \frac{{\mu_{2}^{{}} \beta \left( {e^{ - \lambda b - \lambda L} - e^{\lambda b} } \right)}}{{2\left( {\alpha^{2} - \lambda^{2} } \right)\lambda^{5} \left( {1 - e^{ - 2\lambda L} } \right)}}} \right. + \frac{{\mu_{3}^{{}} \left( {e^{\lambda b} - e^{ - \lambda b - \lambda L} } \right)}}{{2\lambda^{3} \left( {1 - e^{ - 2\lambda L} } \right)}}\left. { + \frac{{\mu_{4}^{{}} \beta \left( {e^{ - \lambda b - \lambda L} - e^{\lambda b} } \right)}}{{2\lambda^{3} \left( {\alpha^{2} - \lambda^{2} } \right)\left( {1 - e^{ - 2\lambda L} } \right)}}} \right\}\left( {e^{ - \lambda x} - e^{ - \lambda L} e^{\lambda x} } \right) \\ & \quad + \frac{{\mu_{2}^{{}} \beta P\left( {e^{\alpha L + \alpha b} - e^{ - \alpha b} } \right)}}{{2\left( {\alpha^{2} - \lambda^{2} } \right)\alpha^{5} \left( {e^{2\alpha L} - 1} \right)}}\left( {e^{\alpha L - \alpha x} - e^{\alpha x} } \right) - \frac{{\mu_{1}^{{}} P\left( {L + 2b} \right)}}{{24\lambda^{2} L}}\left( {3Lx^{2} - 2x^{3} } \right) + \frac{{\mu_{4}^{{}} \beta P\left( {e^{\alpha L + \alpha b} - e^{ - \alpha b} } \right)}}{{2\left( {\alpha^{2} - \lambda^{2} } \right)\alpha^{3} \left( {e^{2\alpha L} - 1} \right)}}\left( {e^{\alpha L - \alpha x} - e^{\alpha x} } \right) - \frac{{\mu_{2}^{{}} \beta P\left( {L + 2b} \right)}}{{24\alpha_{t}^{2} \alpha^{2} L}}\left( {2x^{3} - 3Lx^{2} } \right) \\ & \quad \left[ {\frac{{\mu_{1}^{{}} P}}{{\lambda^{2} L}}\left( {\frac{b}{{\lambda^{2} }} + \frac{L}{{2\lambda^{2} }} + \frac{{bL^{2} }}{12} + \frac{{b^{3} }}{6} + \frac{{b^{2} L}}{4}} \right) - \frac{{\mu_{2}^{{}} \beta P}}{{\lambda^{2} \alpha^{2} L}}\left( {\frac{{bL^{2} }}{12} + \frac{{b^{3} }}{6} + \frac{{Lb^{2} }}{4}} \right) - \frac{{\left( {\alpha^{2} + \lambda^{2} } \right)\mu_{2}^{{}} \beta P\left( {2b + L} \right)}}{{2\alpha^{4} \lambda^{4} L}} + \frac{{\left( {\mu_{3}^{{}} \alpha^{2} - \mu_{4}^{{}} \beta } \right)P}}{{2\lambda^{2} \alpha^{2} L}}\left( {2b + L} \right)} \right]x \\ & \quad - \frac{{\mu_{1}^{{}} P}}{{2\lambda^{2} }}\left( {\frac{b}{{\lambda^{2} }} + \frac{L}{{2\lambda^{2} }} + \frac{{b^{3} }}{6} + \frac{{b^{2} L}}{4} - \frac{{L^{3} }}{24}} \right)\frac{{\left( {\alpha^{2} + \lambda^{2} } \right)\mu_{2}^{{}} \beta PL\left( {2b + L} \right)}}{{4\alpha^{4} \lambda^{4} L}} + \frac{{\mu_{2}^{{}} \beta P}}{{2\lambda^{2} \alpha^{2} }}\left( {\frac{{b^{3} }}{6} + \frac{{Lb^{2} }}{4} - \frac{{L^{3} }}{24}} \right) - \frac{{\left( {\mu_{3}^{{}} \alpha^{2} - \mu_{4}^{{}} \beta } \right)PL}}{{4\lambda^{2} \alpha^{2} L}}\left( {2b + L} \right) \\ \end{aligned} $$

### Design parameter analysis

#### Load

**Figure 6:**
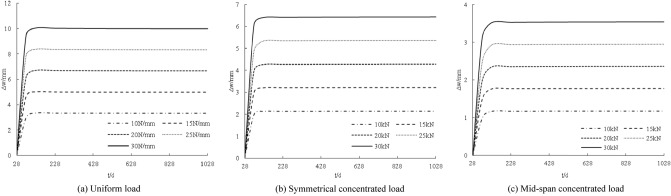
Influence of loading under creep on displacement increment.

The distribution curve of the deformation increment with age under different loads is obtained by a calculation, as shown in Fig. 6. The deformation increments are distributed non-linearly. The deformation increments for 28 days are all zero. With the increase in age, the deformation increments gradually increase. The increase is larger within 100 days, and the deformation increments are basically unchanged from 100 to 1028 days. The deformation increment increases with increasing load. The larger the load, the steeper the deformation increment curve; for every 5 N/mm (5 kN) increase in the load, the deformation increment increases by approximately 1.7 mm, 1.1 mm, and 0.6 mm.

#### Connection stiffness

**Figure 7:**
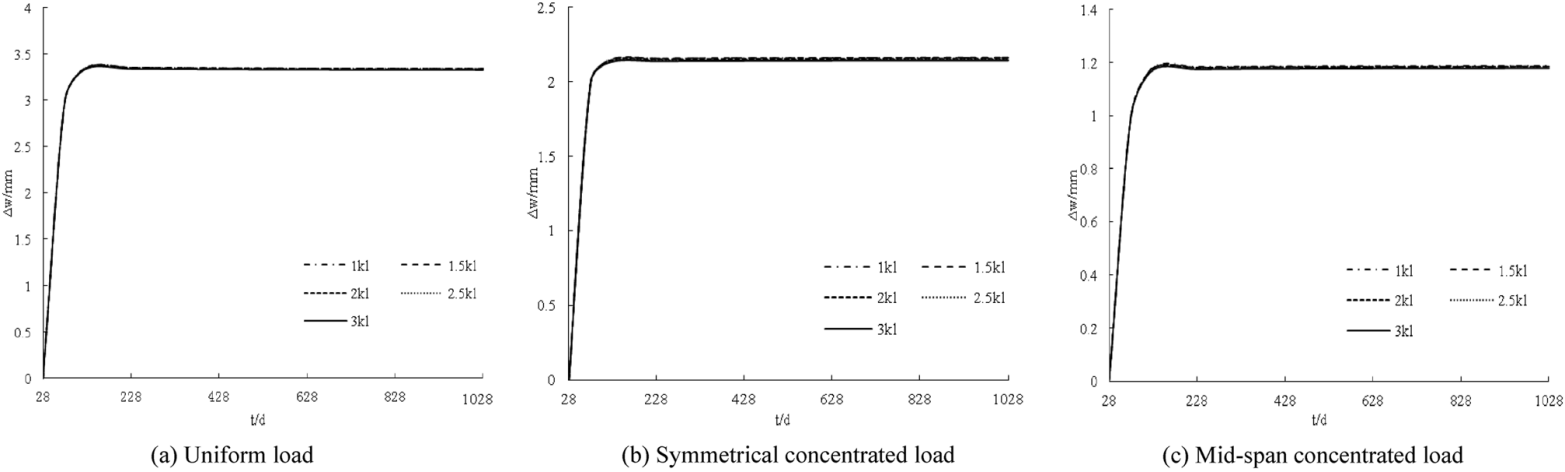
Coupling influence of stiffness under creep on displacement increment.

The distribution curve of the deformation increment with age under different stiffness conditions is shown in Fig. 7. In general, the deformation increment decreases with increasing connection stiffness, but the magnitude of the decrease is minimal, indicating that the connection stiffness is very important to the deformation increment of the component. This impact is not obvious.

## Conclusion

In this study, elastic and energy methods are used to calculate the interfacial slip, axial force, and deformation increment in steel and concrete composite beams strengthened by CFRP sheeting under creep. The conclusions are summarized as follows:

1. The influence of the design parameters on the mechanical properties of the interface was analyzed. The calculation results show that the formula is correct and can be used to calculate the interface slip between the steel beam and the concrete slab reinforced by CFRP sheeting under the action of concrete creep. Based on the correct calculation formulas, the calculation formulas for interface slip, axial force, and deformation increment are derived.
2. Under the action of concrete creep, the slip amount, axial force, and deformation increment between the steel beam and the concrete slab strengthened by CFRP sheeting increase with increasing load. The greater the increase in the connection stiffness, the smaller the increase in the axial force increment.
3. The calculation results show that the increment of interface slip, axial force, and deformation are zero on the 28th day. As the age increases, the increment of interface slip, axial force, and deformation gradually increase; the increase is large in the first 100 days, and basically unchanged from 100 to 1028 days.
4. The calculation results also show that when the load is increased by 5 N/mm (5 kN), the slip increment increases by approximately 0.004 mm, 0.002 mm, and 0.002 mm, and the axial force increment increases by approximately 19.4 kN, 15.9 kN, and 16.1 kN. The deformation increment increases by approximately 1.7 mm, 1.1 mm, and 0.6 mm.
5. When the stiffness increases by one step, the change in the slip increment gradually decreases, and the axial force increases with an increase in connection stiffness. The larger the increase in connection stiffness, the smaller the increase in the axial force increment; the change in the deformation increment with increasing connection stiffness is minimal.
6. The theoretical derivation formula in this study is based on a series of assumptions and ignores the influence of some factors. Various factors should be considered in further research.

## Data Availability

Some or all data, models, or code generated or used during the study are available from the corresponding author by request.
